# Demise of nociceptive Schwann cells causes nerve retraction and pain hyperalgesia

**DOI:** 10.1097/j.pain.0000000000002169

**Published:** 2021-01-22

**Authors:** Puneet Rinwa, Laura Calvo-Enrique, Ming-Dong Zhang, Jens Randel Nyengaard, Páll Karlsson, Patrik Ernfors

**Affiliations:** a Department of Medical Biochemistry and Biophysics, Division of Molecular Neurobiology, Karolinska Institutet, Stockholm, Sweden; b Department of Clinical Medicine—Core Centre for Molecular Morphology, Section for Stereology and Microscopy, Aarhus University, Aarhus, Denmark; c Centre for Stochastic Geometry and Advanced Bioimaging, Aarhus University Hospital, Aarhus, Denmark; d Danish Pain Research Center, Aarhus University, Aarhus, Denmark

**Keywords:** Nociceptive Schwann cells, Nerve retraction, Pain hyperalgesia, Peripheral neuropathy

## Abstract

Recent findings indicate that nociceptive nerves are not “free”, but similar to touch and pressure sensitive nerves, terminate in an end-organ in mice. This sensory structure consists of the nociceptive nerves and specialized nociceptive Schwann cells forming a mesh-like organ in subepidermis with pain transduction initiated at both these cellular constituents. The intimate relation of nociceptive nerves with nociceptive Schwann cells in mice raises the question whether defects in nociceptive Schwann cells can by itself contribute to pain hyperalgesia, nerve retraction, and peripheral neuropathy. We therefore examined the existence of nociceptive Schwann cells in human skin and their possible contribution to neuropathy and pain hyperalgesia in mouse models. Similar to mouse, human skin contains SOX10^+^/S100B^+^/AQP1^+^ Schwann cells in the subepidermal border that have extensive processes, which are intimately associated with nociceptive nerves projecting into epidermis. The ablation of nociceptive Schwann cells in mice resulted in nerve retraction and mechanical, cold, and heat hyperalgesia. Conversely, ablating the nociceptive nerves led to a retraction of epidermal Schwann cell processes, changes in nociceptive Schwann cell soma morphology, heat analgesia, and mechanical hyperalgesia. Our results provide evidence for a nociceptive sensory end-organ in the human skin and using animal models highlight the interdependence of the nerve and the nociceptive Schwann cell. Finally, we show that demise of nociceptive Schwann cells is sufficient to cause neuropathic-like pain in the mouse.

## Abstract 2

The role of nociceptive Schwann cells for chronic pain is not known. We find that their demise is sufficient to cause neuropathy and neuropathic pain.

## 1. Introduction

Sensory end-organs located in the skin are responsible for cutaneous touch and pressure sensation and are made up of nonneuronal cells and sensory nerves. Merkel cells and innervating sensory afferents are both mechanosensitive and contribute to pressure sensation by responding to mechanical force applied to the skin. As such, Merkel cells represent a sensory end-organ receptor cell type, which signal to the nerve and contribute to activation of the low-threshold mechanoreceptors (LTMR).^24,25,39^ In contrast to the Merkel cell-neurite complex end-organ, Pacinian corpuscles, Meissner corpuscles, Ruffini corpuscles, and lanceolate endings of hair follicles are made up of terminal Schwann cells, which together with the LTMR nerves build the sensory organ structures.^45^ Within these, the Schwann cells are believed to provide an important structural function by anchoring LTMR nerves to the surrounding dermis and epidermis. However, unlike all the above touch-sensitive nerves that associate with end-organs, nociceptors have traditionally been considered as special among cutaneous sensory nerves, in that they terminate as “free” nerve endings in the absence of any apparent end-organ. Because of this, the main focus for understanding and treating chronic pain disorders has been on the nociceptive neurons.

However, it was recently reported in the mouse that nociceptive nerves, like most of the LTMRs, are associated with specialized Schwann cells, which together form a mesh-like network in the border between the dermis and epidermis with radial processes abutting to unmyelinated nerves extending into the epidermis.^1^ These results show that nociceptors associate with molecularly and morphologically specialized Schwann cells that together form a glioneural end-organ structure. The nociceptive Schwann cells are inherently mechanosensitive, act as sensory receptor cells, and contribute to the initiation of pain.^1^ Nociceptive nerves are generally unmyelinated and therefore associate with Remak Schwann cells that have supportive functions.^13,27^ The close association between the nerve and the nociceptive Schwann cells open the question on the interdependence of the nerve and nociceptive Schwann cell, and whether dysfunction of the nociceptive Schwann cells can result in nerve sensitization and hyperalgesia and, by this, participate in chronic pain disorders. Therefore, this study aimed to examine the existence of nociceptive Schwann cells in humans and to determine whether they contribute to axon integrity and pain sensitization in a well-established animal model of neuropathic pain.

## 2. Materials and methods

### 2.1. Mouse strains

All animal work was permitted by the Ethical Committee on Animal Experiments (Stockholm North committee) and conducted according to The Swedish Animal Agency's Provisions and Guidelines for Animals Experimentation recommendations. Mice of both sexes and from mixed background were used in this study. Animals were kept in cages in groups, with food and water ad libitum, under 12-hour light–dark cycle conditions. Sox10^CreERT2^ mouse strain has been previously described.^21^ Rosa26R^tdTomato^ (Ai14, stock number 007914) and Rosa26R^DTA^ (Ai40D, stock number 021188) were ordered from The Jackson Laboratory (Bar Harbor, ME). Sox10^CreERT2^ mice were coupled to R26R^DTA^ and R26R^TOM^ mice for behavioral and functional experiments. The resulting strains from the crosses were the following: Sox10CreERT2/+; R26RTOM/+ (abbreviated Sox10-TOM) and Sox10CreERT2/+; R26RDTA/DTA (abbreviated Sox10-DTA). The animals used had a mixed genetic background (C57BL6, 6J, 6N).

Tamoxifen (Sigma Aldrich Sweden, Stockholm, Sweden, T5648) was dissolved in corn oil (Sigma, 8267) at a concentration of 20 mg/mL and delivered by intraperitoneal (i.p.) injection to adult for 2 consecutive days (200 mg/kg). Controls and test mice received tamoxifen injections. Behavioral tests were performed 7 days after the last tamoxifen injection.

Resiniferatoxin (Sigma-Aldrich Sweden AB, 14364) was dissolved in the vehicle (10% Tween 80 and 10% ethanol in normal saline) at a concentration of 5 µg/mL and delivered by intraperitoneal (i.p.) injection to adult mice for one day (50 µg/kg). This resiniferatoxin model is largely reversible in terms of behavioral and morphological alterations^6,15^

Hydroxy-tamoxifen (OH-Tx) (Sigma-Aldrich Sweden AB, 66459) was dissolved in 100% ethanol at a concentration of 10 mg/mL and delivered by cutaneous painting to the glabrous skin of adult mice for 3 consecutive days (1 mg/mouse, 200 µL/mouse). Mice were under isoflurane anesthesia for 10 minutes during painting. Animals paw was cleaned thoroughly with wet wipe to remove the OH-Tx residues and prevent animal from licking and orally ingesting the drug.

### 2.2. Tissue preparation

Adult mice were sacrificed with isoflurane overdose and transcardially perfused with 20 mL PBS and 20 mL 4% paraformaldehyde (PFA, Roth, #P087.3). Paws, sciatic or medial plantar nerve, and lumbar dorsal root ganglia (DRG) were then collected and postfixed in PFA (1 hours for DRG and nerves, 2 hours for paws and skin) at 4°C, washed 3 times with PBS, and cryoprotected by incubating at 4°C in 30% sucrose in PBS with 0.02% sodium azide for 24 hours. Plantar/palmar skin of each paw was then dissected out, and tissue was embedded in optimum cutting temperature compound (Tissue-Tek, Sakura Finetek Europe B.V., Alphen aan den Rijn, the Netherlands) and frozen at −20°C. Tissue samples were sectioned at 14 or 20 µm thickness and conserved at −20°C until further use. For electron microscopy, plantar skin of hind paws were dissected out and fixed by immersion in 2% glutaraldehyde and 1% paraformaldehyde for transmission electron microscopy.

Healthy human skin punch biopsies were harvested from the lower leg/ankle region, immersion-fixed in 4% PFA (Roth) or 2% PFA with picric acid (Zamboni Fixative) for 1 hour on ice, and cryoprotected in 10% sucrose (in 0.1M PB) containing 0.01% sodium azide (Sigma) and 0.02% bacitracin (Sigma) for 2 days.^17,18^ Skin biopsies were embedded with optimum cutting temperature compound, frozen, and cut in a cryostat (ThermoFisher Scientific, Waltham, MA,) at 20 and 30 µm. Sections were stored at −20°C until further use. This human part of the study was performed in accordance with The Helsinki Declaration II. The human skin biopsies originate from an ongoing study at Aarhus University, Denmark, which is approved by the Regional Ethics Committee (case no. 1-10-72-103-19) and by the Danish Data Protection Agency. All participants provided their informed voluntary consent before study start.

### 2.3. Immunohistochemistry

Thawed sections were air-dried for at least one hour at room temperature (RT). Cultured cells were fixed with 4% PFA for 20 minutes and then washed 3 times with PBS, and immunocytochemistry was performed in the same way as for skin sections. Antigen retrieval was performed for SOX10, PGP9.5, and S100beta staining in the skin. For that, sections were immersed in 1x Target Retrieval Solution (Dako, Agilent Technologies, Santa Clara, CA, #S1699) in water for 20 minutes, preheated at 80°C. Sections were then washed 2 times in PBS and incubated in blocking solution (5% normal donkey serum [Jackson Immuno Research, #017-000-121], 2% Bovine Serum Albumin [Sigma, #A7906], 0.3% Triton X-100 in PBS) for 1hour before applying primary antibodies overnight at 4°C. The following primary antibodies (diluted in the blocking solution) were used: rabbit anti–PGP9.5 (1:1000, AbD Serotec, Bio-Rad Laboratories, Hercules, CA, #7863-0504), goat anti-SOX10 (1:200, Santa Cruz Biotechnologies, Santa Cruz, CA, #sc-17342), rabbit anti-VR1 (1:50, Calbiochem, Sigma-Aldrich Sweden, #PC420), and mouse anti-S100beta (1:500, Abcam, #Ab4066). For detection of the primary antibodies, secondary antibodies raised in donkey and conjugated with Alexa-488, Alexa-555, and Alexa-647 fluorophores were used (1:1000, Molecular Probes; Thermo Fisher Scientific) for 1hour at RT. DAPI staining (1 mg/mL, Thermo Fisher Scientific, #D1306) was performed at the same time as secondary antibodies. Sections were then washed 3 times with PBS and mounted using fluorescent mounting medium for imaging (Dako, #S3023). For staining in human skin biopsies, sections were dried at RT for 30 minutes, and incubated with goat anti-Sox10 (1:500, Santa Cruz) or rabbit ant-PGP9.5 (1:1000, UltraClone, RA95101) antibody overnight at 4°C; sections were developed and visualized with TSA Plus kit (PerkinElmer Denmark, Skovlunde, Denmark) according to manufacturer's protocol. Double- and triple-labelling were performed with combinations of rabbit anti-S100b (1:400, Dako), mouse anti-MART-1 (1:40, BioLegend, M2-7C10), goat anti-Sox10 (1:100, Santa Cruz), or mouse anti-AQP1 (1:50, Santa Cruz, #sc-25287) at 4°C for 2 days incubation. Immunoreactivities were visualized with secondary antibodies conjugated with Alexa-555 and Alexa-647 fluorophores (1:500, Molecular Probes; Thermo Fisher Scientific) at RT for 2 hours. DAPI (Thermo Fisher Scientific) was added to visualize nucleus. Coverslips were mounted with mounting medium from Dako. Images were acquired using Zeiss LSM700 confocal microscope equipped with 20× and 40× objectives. Images were acquired in the .lsm format and processed with ImageJ. Representative images are maximum-intensity projections of Z-stacks taken at 1-μm intervals.

### 2.4. Electron microscopy

For transmission electron microscopy, skin samples were fixed by immersion in 2% glutaraldehyde and 1% paraformaldehyde in 0.1 M PB for 24 hours at 4°C, washed with 0.1M PB, and postfixed in 2% osmium tetroxide (OsO4) in 0.1 M PB for 2 hours at 4°C. Samples were then dehydrated by incubation in ethanol, followed by acetone before embedding in epoxy resin LX-112 (Ladd, Burlington, VT). Semithin sections were first made and stained with toluidine blue for light microscopy analysis. Ultrathin sections were made with Leica Ultracut UCT (Leica, Vienna, Austria), contrasted with uranyl and examined in a Tecnai 10 transmission electron microscope at 80 kV (FEI Company, Hillsboro, OR). Digital images were made using a MegaViewIII digital camera (soft imaging system, GmbH, Münster, Germany). EM microscopy has been done in the center for high-resolution electron microscopy core facility (Department of Bioscience and Nutrition, Karolinska Institutet).

### 2.5. Behavior studies

All behavioral tests were performed on adult mice (2-4 months) from both sexes (males and females were randomly divided into different groups so that every group has similar number of animals from each sex) and their corresponding littermates were used as controls (expressing the drivers but not reporters). The examiners were blind to genotype/treatment when undertaking rodent behavioural tests.

### 2.6. Behavior tests

#### 2.6.1. Mechanical threshold

After a resting period on a mesh floor, the plantar surface of the hind paws were stimulated with a series of calibrated monofilaments (von Frey hairs; Stoelting, IL) with increasing force until the desired responses were elicited. Each filament was applied 5 times. The withdrawal threshold was defined as the force at which the animal withdrew the paw at least 3 out of the 5 trials.

#### 2.6.2. Hargreaves test

A radiant heat source (IITC, Woodland Hills, CA) was aimed at the plantar surface of the hind paw through a glass surface. Readout was the withdrawal latency of the stimulated paw.

#### 2.6.3. Acetone evaporation assay

The acetone evaporation assay was performed using the scoring method with minor modifications.^5,7^ Briefly, mice were first acclimated on a mesh surface. Then, the plantar skin of the hind paw was gently contacted with an acetone drop formed at the tip of a 1-mL syringe. Response to acetone cooling effect was scored from 0 to 5 according to the following scale: 0 = no response, 1 = brief lift, sniff, flick, or startle; 2 = jumping, paw shaking; 3 = multiple lifts, paw lick; 4 = prolonged paw lifting, licking, shaking, or jumping; 5 = paw guarding. Mice were tested 5 times on each paw with a waiting time of 2 minutes.

#### 2.6.4. Pinprick test

A 27-gauge needle was gently applied to the plantar surface of the hind paw without penetrating the skin. A score system was used according to the extent of the response. 0 = no response; 1 = move, look around to see what happened; 2 = brief quick lift or withdrawal or remove away of hind paw; 3 = brief quick shakes of hind paw, or jumps; 4 = high frequency of shaking, licking, flinching, or guarding.

### 2.7. Fluorescence activated cell sorting and quantitative polymerase chain reaction

Terminal Schwann cells were obtained from glabrous skin of Sox10-TOM+ pups (P11-P14, after tamoxifen administration, 2 mg/20 g body weight). Briefly, paws were quickly collected in ice-cold HBSS medium (Thermo Fisher Scientific, #14170112) containing 100 U/mL penicillin and 100 μg/mL streptomycin (supplied as a mix, Thermo Fisher Scientific, #15140122). Plantar skin was then dissected out and nerves and other tissues were removed. Skin was incubated in HBSS containing 4 mg/mL of collagenase/dispase (Sigma-Aldrich, cat.11097113001) for 30 minutes at 37°C. Epidermis was then removed and the dermis, after careful removal of footpads, was cut in small pieces and incubated with collagenase/elastase (Worthington. Cat. LK002066) 4 mg/mL in HBSS for 30 minutes at 37°C. DNAse I was added (Worthington. Cat. LK003170) to a final concentration of 1 mg/mL before mechanical dissociation with fire-polished Pasteur pipettes coated previously with 1% bovine serum albumin in PBS. The cell suspension was slowly filtered through 40-μm-pore size cell strainer and centrifuged at 400*g* for 6 minutes. Tomato-positive cells were directly sorted into lysis buffer (350 per 100 μL sorted cells) with a fluorescence-activated cell sorting Aria III (BD Bioscience, Franklin Lakes, NJ) at the Biomedicum Flow Cytometry Core Facility (Karolinska Institutet, Solna) for quantitative polymerase chain reaction (qPCR) or plated in coverslips for immunohistochemistry analysis. Dorsal root ganglia were dissected from young adult mice. RNA was isolated using RNeasy microkit (Qiagen) following manufacturer's instructions. Complementary deoxyribonucleic acid was synthetized using RT^2^ First Strand (Qiagen) following manufacturer's instructions.

Real-time qPCR was performed using Qiagen's RT^2^ Profiler PCR array mouse neuronal ion channels (#PAMM-036Z) and RT2 SYBR Green Rox qPCR mastermix (#330524). PCR was performed with StepOnePlus (Applied Biosystems, Waltham, MA). Data analyses were conducted using Ct red out and were normalized to the expression of the following housekeeping genes: Actb, B2m, Gapdh, Gusb, and Hsp90ab1.

### 2.8. Quantification and statistics

Data were analyzed using GraphPad Prism 5 and expressed as mean ± standard error of the mean (SEM). Individual tests and their respective post hos test and sampling size are mentioned in the figure legends. Degree of significance was indicated in each comparison: *P ≤ 0.05; **P ≤ 0.01; ***P ≤ 0.001; ****P ≤ 0.0001. No statistical methods were used to predetermine sample size, but our sample sizes are similar to those generally used in the field.

### 2.9. Quantifying intraepidermal nerve fibres, Schwann cell processes, and Schwann cells

Randomly chosen tissue sections were quantified from each animal. Quantification of intraepidermal nerve fibres (IENFs) was performed according to Lauria et al.^22^ Only single IENFs crossing the dermal–epidermal junction were counted and those with branching points within the epidermis were counted as a single fibre. For fibres with branching points in the dermis, each branch was counted as a single IENF. The length of the lower margin of the stratum corneum was defined as the epidermal length and determined with Image J software. IENF density was defined as the counted IENFs divided by the epidermal length (fibres/mm). Same rule was applied to the Schwann cell processes. Schwann cells were counted based on SOX10 staining (glial cell nucleus) at the intraepidermal border and counted as number per epidermal length. Male and female control Sox10^Tom^ reporter line was used to test if OH-Tx has any effect on Schwann cells or intraepidermal fibers. OH-Tx was painted onto the glabrous skin of hind paw and Sox10+ nociceptive Schwann cells and PGP9.5-positive intraepidermal nerve fibers were quantified 7 days later. No difference in Schwann cell numbers of IENFs were observed between the experimental control groups.

## 3. Data availability

Data are available upon request.

## 4. Results

### 4.1. Nociceptive Schwann cells are present both in mouse and human skin

**Figure 1.**
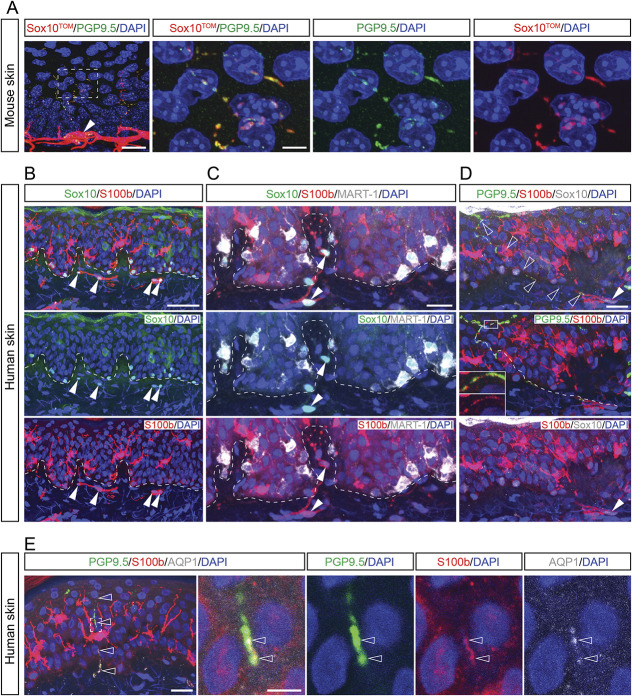
The nociceptive glioneural sensory organ in human skin. (A) Labeling of PGP9.5 (green, nerves) with Sox10^TOM^ (red, nociceptive Schwann cells) in Sox10-TOM mouse epidermis/dermis skin. The processes of nociceptive Schwann cells climbing with nerve fibers from the bottom layer of epidermis. Scale bar: 20 μm in (A), and 5 μm in the inset from (A). (B and C) Sox10 labels cells along the bottom layer of epidermis and along the junction between dermis and epidermis of human skin. S100b labels dendritic cells, nociceptive Schwann cells, and very weakly labels melanocytes along the junction between dermis and epidermis. MART-1 labels melanocytes in human skin. Arrowheads indicate the nociceptive Schwann cells (Sox10^+^S100^+^MART-1^−^). Dash line labels the border between epidermis and dermis. Scale bar: 50 μm in (B) and 20 μm in (C). (D) The nociceptive Schwann cell (indicated by filled arrowhead) located in the epidermis/dermis junction sends process into the epidermis (unfilled arrowheads) along the nerve fiber. The dashed line shows the path of process from the nociceptive Schwann cell. The inset highlights the close contact of nociceptive Schwann cell and nerve fibers. Scale bar: 20um. (E) Triple labeling of PGP 9.5 (green), S100b (red), and AQP1 (gray) in human dermis/epidermis skin. The enlarged area from the inset in (E) shows the close contact of nociceptive Schwann cell process and nerve fiber in epidermis (indicated by unfilled arrowheads). The enlarged images were taken from 1-μm-thick tissue. Scale bar: 20 μm in (E), 5 μm for the inset.

We bred the neural-crest and glia-specific inducible Sox10-CreERT2 mouse line to the R26R^tdTOMATO^ (R26R^TOM^) reporter line generating Sox10^CreERT2/+^; R26R^TOM/+^ (abbreviated Sox10^TOM^) mice and administered tamoxifen to the adult animal to visualize the nociceptive glioneural complex. Similar to previous results,^1^ nociceptive Schwann cells formed a mesh-like network in the epidermal–dermal border of the glabrous skin of mice with radial processes closely associated to nerves extending into the epidermis (Fig. 1A). We thereafter obtained human skin biopsies from healthy volunteers and stained for SOX10 and S100B that are known markers for mouse nociceptive Schwann cells. Labeled SOX10^+^ nuclei were found in dermis close to the epidermal border (Fig. 1B, arrowheads), as well as cells in the basal layer of epidermis, whereas S100B-labeled cells in the subepidermal border, basal layer of epidermis, and scattered cells with dendritic morphology in epidermis. SOX10^+^ cells at the basal layer of epidermis stained weakly also for S100B and were MART-1^+^ melanocytes, whereas the SOX10^-^ nuclei and S100B^+^ cells with dendritic morphology scattered in the spinous layer of epidermis were Langerhans cells (Fig. 1C).^3^ Although neither melanocytes nor Langerhans cells associated with PGP9.5^+^ nerves, the subepidermal SOX10^+^:S100B^+^ Schwann cells associated with nerves and displayed S100B^+^ Schwann cell processes abutted to PGP9.5^+^ nerves extending into epidermis (Fig. 1D). This conclusion was confirmed by staining for AQP1, a marker expressed in nociceptive Schwann cells^1^ but not in melanocytes or Langerhans cells (Fig. 1E). These results evidence the existence of a nociceptive sensory end-organ in human skin.

### 4.2. Loss of nociceptive Schwann cells affects pain behavior

**Figure 2.**
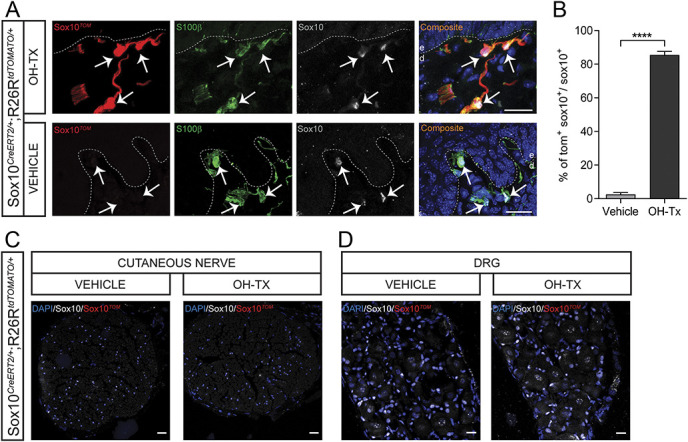
Recombination in the glabrous skin, cutaneous nerve, and dorsal root ganglia after hydroxy tamoxifen painting in Sox10-TOM mice. (A) Recombination in the glabrous skin of vehicle-painted and hydroxy tamoxifen (OH-Tx)-painted paw in Sox10-TOM mice. Immunohistochemistry for TOMATO, S100β, and Sox10. Recombination is evident in OH-Tx-painted paw, whereas no recombination at all was observed in vehicle-painted paw. Arrows indicate Schwann cell nucleus. Dashed line indicates dermal–epidermal border. Scale bar: 20 µm. (B) Quantification of recombination in glabrous skin as represented by % of TOM^+^SOX10^+^ cells to total SOX10+ cells in vehicle-painted and OH-Tx-painted paw (n = 165 cells in vehicle treatment and n = 170 cells in OH-Tx, n = 2 animals per treatment). Two-tailed unpaired *t* test with Welch correction. *P* < 0.0001. Data are presented as mean ± standard error of the mean. (C) Recombination in the median palmar nerve of vehicle-painted and OH-Tx-painted paw in Sox10-TOM mice. Immunohistochemistry for TOMATO and Sox10. No recombination at all was observed in both OH-Tx- and vehicle-painted paw. Scale bar: 20 µm. (D) Recombination in the dorsal root ganglion of vehicle-painted and OH-Tx-painted paw in Sox10-TOM mice. Immunohistochemistry for TOMATO and Sox10. No recombination at all was observed in both OH-Tx- and vehicle-painted paws. Scale bar: 20 µm.

To study the effects of dysfunctional nociceptive Schwann cells in a mouse model, we first developed an intersectional strategy to exclusively activate the CreERT2 recombinase in cutaneous but not in other Schwann cells. For establishing this strategy, we first made use of the Sox10^Tom^ reporter line. To develop a strategy affecting only the cutaneous Schwann cells, we painted 4-hydroxy tamoxifen (OH-Tx) onto the glabrous skin of hind paw. Recombination in Schwann cells was analyzed by Tomato fluorescence and staining with the Schwann cell markers S100B and SOX10, which revealed an efficient recombination in cutaneous dermal Schwann cells including Schwann cells in the dermal–epidermal border (92%) (Figs. 2A and B). The specificity of the strategy for cutaneous Schwann cells was ascertained by analyzing the median palmar nerve and dorsal root ganglion, neither of which contained any Tomato^+^ cells (Figs. 2C and D). We therefore conclude that this method allows for addressing the role of nociceptive Schwann cells for cutaneous neuropathy and neuropathic pain.

**Figure 3.**
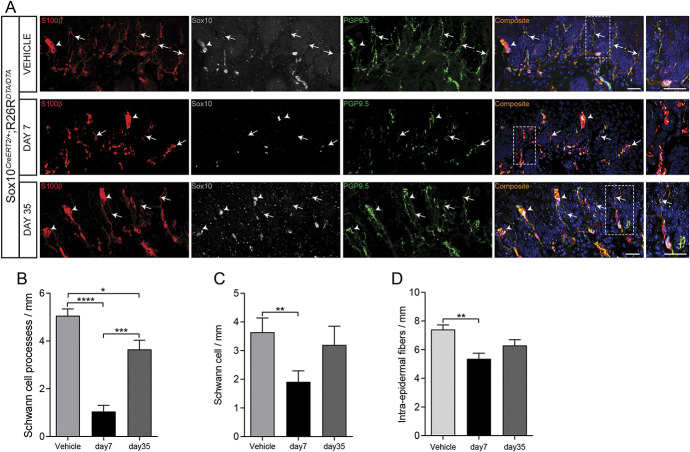
Depletion of intraepidermal fibers and terminal Schwann cells after hydroxy tamoxifen painting in the glabrous skin of Sox10-DTA mice. (A) The intraepidermal fibers in the glabrous skin of hydroxy tamoxifen (OH-Tx)-painted paw gets depleted on day 7 but partially reappear on day 35 as compared to vehicle treatment in Sox10-DTA mice. Immunohistochemistry for S100β, Sox10, and PGP9.5. Arrows indicate intraepidermal small fibers. Arrowheads indicate Meissner corpuscle. Dashed line indicates dermal–epidermal border. Enlargements of the fibers are boxed in the side magnification images. Scale bar: 20 µm. (B) Quantification of Schwann cell processes as represented by Schwann cell processes per mm in vehicle-painted and day 7 and day 35 of OH-Tx-painted paw (n = 3). Data are presented as mean ± standard error of the mean (SEM). Two-tailed unpaired *t* test with Welch correction for vehicle vs day 7 *P* < 0.0001 (****), for vehicle vs day 35 *P* = 0.0157 (*), and for day 7 vs day 35 *P* = 0.0003 (***). (C) Quantification of number of Schwann cells as represented by Schwann cells per mm in vehicle-painted and day 7 and day 35 of OH-Tx-painted paw (n = 3). Data are presented as mean ± SEM. Two-tailed unpaired *t* test with Welch correction for vehicle vs day 7 *P* = 0.0025 (**). (D) Quantification of intraepidermal fibers as represented by intraepidermal fibers per mm in vehicle-painted and day 7 and day 35 of OH-Tx-painted paw (n = 3). Data are presented as mean ± SEM. Two-tailed unpaired *t* test with Welch correction for vehicle vs day 7 *P* = 0.0025 (**).

To ablate the nociceptive Schwann cells, Sox10Cre^ERT2^ mice were crossed to mice carrying conditional expression construct for diphtheria toxin that can be activated upon Cre‐mediated recombination^19^ to generate Sox10Cre^ERT2/+^; R26R^DTA/DTA^ mice. OH-Tx painting of these mice led at 7 days to a marked loss of intraepidermal Schwann cell processes, a reduction of Sox10^+^ cells, and also a reduction of intraepidermal nerve fibers, which was largely recovered at day 35 (Fig. 3).

**Figure 4.**
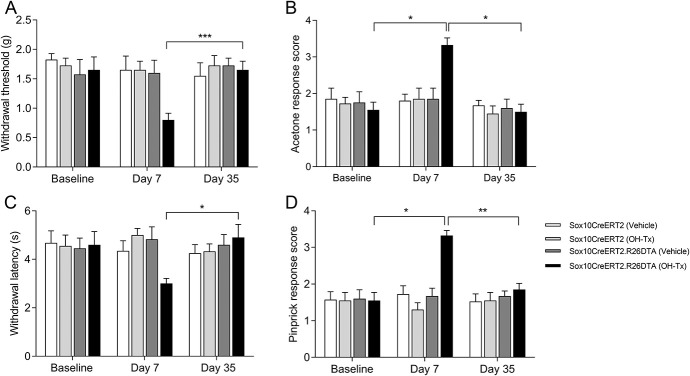
Behavior studies on Sox10-DTA mice after hydroxy tamoxifen (OH-Tx) painting. (A) Behavioral response to mechanical stimuli using von Frey filaments in Sox10Cre^ERT2/+^ and Sox10Cre^ERT2/+^; R26R^DTA/DTA^ mice with vehicle and OH-Tx treatments at day 0 (Baseline), day 7, and day 35 (n = 4/group) mice. There was only significant difference observed in Sox10Cre^ERT2/+^; R26R^DTA/DTA^ (Sox10CreERT2DTA) mice treated with OH-Tx. Tukey multiple comparisons test. *P* = 0.001 (***) day 7 vs day 35 compared to vehicle treatment on day 7 ** *P* = 0.0132, 2-tailed Wilcoxon matched-pairs signed rank test. Data are presented as mean ± standard error of the mean (SEM). (B) Behavioral response to cold stimuli using acetone evaporation test score in Sox10Cre^ERT2/+^ and Sox10Cre^ERT2/+^; R26R^DTA/DTA^ mice with vehicle and OH-Tx treatments at day 0 (Baseline), day 7 and day 35 (n = 4/group) mice. There was only significant difference observed in Sox10Cre^ERT2/+^; R26R^DTA/DTA^ mice treated with OH-Tx. Tukey multiple comparisons test. *P* = 0.0255 (*) Baseline vs day 7 and *P* = 0.0174 (*) day 7 vs day 35. Data are presented as mean ± SEM. (C) Withdrawal latency to radiant heat (Hargreaves assay) in Sox10Cre^ERT2/+^ and Sox10Cre^ERT2/+^; R26R^DTA/DTA^ mice with vehicle and OH-Tx treatments at day 0 (Baseline), day 7, and day 35 (n = 4/group) mice. There was only significant difference observed in Sox10Cre^ERT2/+^; R26R^DTA/DTA^ mice treated with OH-Tx. Tukey multiple comparisons test. *P* = 0.0489 (*) day 7 vs day 35. Data are presented as mean ± SEM. (D) Behavioral response to pinprick in Sox10Cre^ERT2/+^ and Sox10Cre^ERT2/+^; R26R^DTA/DTA^ mice with vehicle and OH-Tx treatments at day 0 (Baseline), day 7, and day 35 (n = 4/group) mice. There was only significant difference observed in Sox10Cre^ERT2/+^; R26R^DTA/DTA^ mice treated with OH-Tx. Tukey multiple comparisons test. *P* = 0.0121 (*) Baseline vs day 7 and *P* = 0.0036 (**) day 7 vs day 35. Data are presented as mean ± SEM.

We next examined if the loss of nociceptive Schwann cells affects pain behavior. Animals were examined before OH-Tx (baseline) and 7 and 35 days after OH-Tx painting of the skin. To exclude unrelated effects caused by genotype and OH-Tx, we analyzed Sox10Cre^ERT2/+^ and Sox10Cre^ERT2/+^; R26R^DTA/DTA^ mice with and without OH-Tx. Sox10Cre^ERT2/+^; R26R^DTA/DTA^ mice 7 days after OH-Tx administration displayed a sensitization to mechanical stimuli (Von Frey), cold (acetone), heat (Hargreaves), and pinprick, but none of the other conditions, genotypes, or timepoints (Fig. 4). Thus, an absence of nociceptive Schwann cells leads to a sensitization to both mechanical and thermal stimuli.

### 4.3. Absence of nociceptors compromises nociceptive Schwann cells

**Figure 5.**
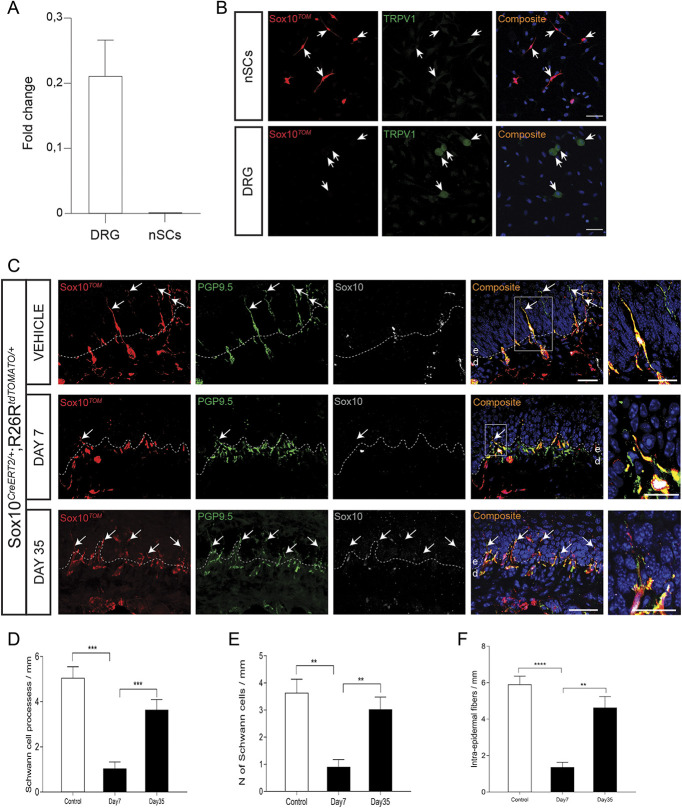
Depletion of intraepidermal fibers and terminal Schwann cells in the glabrous skin of Sox10-TOM mice after resiniferatoxin (RTX) administration. (A) Fold change in TRPV1 expression in dorsal root ganglia (DRG) vs nociceptive Schwann cells (nSCs) measured with quantitative polymerase chain reaction against 5 housekeeping genes. (B) Immunohistochemistry for TRPV1 in cultured nSCs (2 DIV) and in cultured DRG neurons (3DIV) from Sox10TOM animals. Scale bar: 50 μm. Note that only DRG neurons express TRPV1. (C) The intraepidermal fibers in the glabrous skin of RTX-administered mice gets depleted on day 7 but partially comes back on day 35 as compared to Vehicle treatment in Sox10-TOM mice. Immunohistochemistry for TOMATO, Sox10, and PGP9.5. Arrows indicate intraepidermal small fibers. Dashed line indicates dermal–epidermal border. Enlargements of the fibers are boxed in the side magnification images. Scale bar: 20 µm. (D) Quantification of Schwann cell processes as represented by Schwann cell processes per mm in vehicle and, day 7 and day 35 of RTX-administered mice (n = 3).Two-tailed unpaired *t* test with Welch correction for vehicle vs day 7 (*P* = 0.0001; ***) and for day 7 vs day 35 (*P* = 0.001; ***). Data are presented as mean ± standard error of the mean (SEM). (E) Quantification of number of Schwann cells as represented by Schwann cells per mm in vehicle and, day 7 and day 35 of RTX-administered mice (n = 3).Two-tailed unpaired *t* test with Welch correction for vehicle vs day 7 (*P* = 0.0015; **) and for day 7 vs day 35 (*P* = 0.0035; **). Data are presented as mean ± SEM. (F) Quantification of intraepidermal fibers as represented by intraepidermal fibers per mm in vehicle and, day 7 and day 35 of RTX-administered mice (n = 3).Two-tailed unpaired *t* test with Welch correction for vehicle vs day 7 (*P* ≤ 0.0001; ****) and day 7 vs day 35 (*P* = 0.0018; **). Data are presented as mean ± SEM.

**Figure 6.**
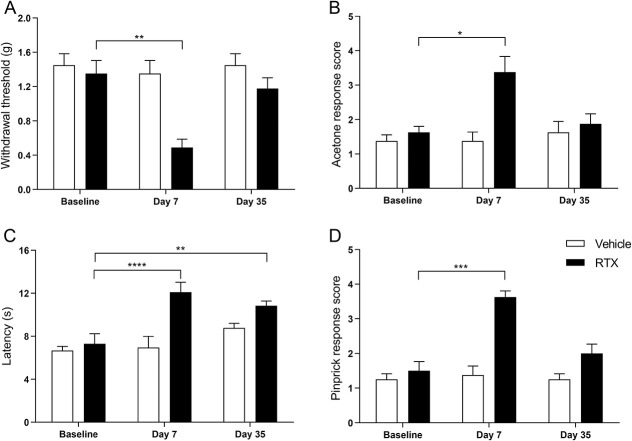
Behavior studies after resiniferatoxin (RTX) administration in the glabrous skin of Sox10-TOM mice. (A) Behavioral response to mechanical stimuli using von Frey filaments in vehicle and RTX treatment at day 0 (Baseline), day 7, and day 35 (n = 8/group) mice. Dunnett multiple comparisons test. Baseline vs day 7 in RTX-treated mice *P* = 0.0046 (**). Data are presented as mean standard error of the mean (SEM). (B) Behavioral response to cold stimuli using acetone evaporation test score in vehicle and RTX treatment at day 0 (Baseline), day 7, and day 35 (n = 4) mice. Dunnett multiple comparisons test. Baseline vs day 7 in RTX-treated mice *P* = 0.0164 (*). Data are presented as mean SEM. (C) Withdrawal latency to radiant heat (Hargreaves assay) in vehicle and RTX treatment at day 0 (Baseline), day 7, and day 35 (n = 4) mice. Dunnett multiple comparisons test. Baseline vs day 7 in RTX-treated mice *P* < 0.0001 (****) and baseline vs day 35 *P* = 0.028 (**). Data are presented as mean SEM. (D) Behavioral response to pinprick in vehicle and RTX treatment at day 0 (Baseline), day 7, and day 35 (n = 4) mice. Dunnett multiple comparisons test. Baseline vs day 7 in RTX-treated mice *P* = 0.0003 (***). Data are presented as mean SEM.

To generate an experimental neuropathy model in which small-diameter sensory nerves are specifically affected without effects on nociceptive Schwann cells, we used the capsaicinoid resiniferatoxin (RTX), a natural substance present in a Moroccan cactus-like plant. RTX is an ultrapotent TRPV1 agonist and hence acts on DRG neurons and their nerve fibers expressing this channel.^28,38,40^ It is a reversible model^6,15^ that depletes mainly the CGRP-positive intraepidermal nerve fibers,^15^ which leads to thermal analgesia and mechanical hyperalgesia.^16,28,34^ Expression of TRPV1 in sensory neurons is well characterized^35,41^; however, it is not known whether it is expressed in nociceptive Schwann cells. To examine expression of TRPV1, we prospectively isolated nociceptive Schwann cells by fluorescence-activated cell sorting and prepared RNA from these and from DRG. Quantitative PCR revealed abundant expression in the DRG, but complete absence in nociceptive Schwann cells (Fig. 5A). This finding was ascertained also by TRPV1 immunohistochemistry of acutely dissociated Sox10^Tom^ nociceptive Schwann cells, which unlike DRG neurons were negative (Fig. 5B). Thus, in the two-cell structure of the nociceptive glioneural complex, only the nerve terminal will be targeted by RTX. We administered 50 μg/kg RTX intraperitoneally in Sox10^TOM^ mice. Nociceptive Schwann cell processes (Sox10^TOM+^), nociceptive Schwann cells (SOX10^+^ cells), and intraepidermal nerve fibers (PGP9.5^+^) were quantified in vehicle-treated mice and RTX-treated mice at day 7 and day 35. RTX-treated mice had a significant reduction of Schwann cell processes, Schwann cells, and intraepidermal fibers at day 7 with a recovery at day 35 (Figs. 5C and D). Thus, there is a mutual dependence between the unmyelinated nerve and the nociceptive Schwann cells, and in the absence of the nerve, nociceptive Schwann cells are compromised. The cutaneous deficits correlated with a sensitization to mechanical stimuli (Von Frey), cold (acetone), and pinprick, while showing analgesia toward radiant heat (Hargreaves) (Fig. 6).

### 4.4. Transmission electron microscopy reveals morphological changes

**Figure 7.**
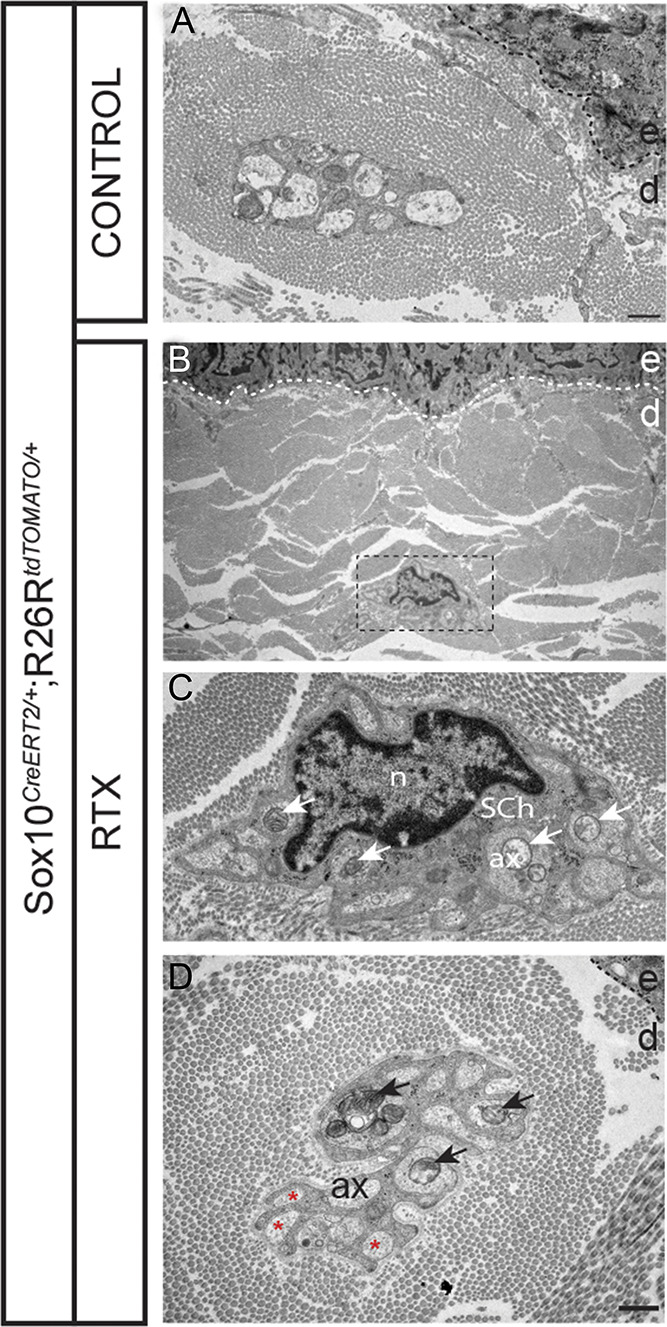
Transmission electron microscopy after resiniferatoxin (RTX) administration in the glabrous skin of Sox10-TOM mice. Transmission electron microscopy of the Schwann cells–small fibers (axons) complex in the glabrous skin. (A) Control treatment with several axons in a remake bundle at the dermal–epidermal border, most of which are fully enveloped by the Schwann cell. Scale bar: 1 µm. (B) RTX treatment with a remake bundle close to the dermal–epidermal border. Scale bar: 1 µm. (C) Remake bundle at higher magnification of boxed area with arrows points to mitochondrial accumulation in the axons. Scale bar: 1 µm. (D) RTX treatment with a remake bundle at the dermal–epidermal border with arrows points to mitochondrial accumulation in the axons, and with asterisk in axons with partial Schwann cell ensheathment. Ax, axon; d, dermis; e, epidermis; n, nucleus, Sch, Schwann cell. Scale bar: 1 µm.

The profound effects of retraction of the unmyelinated intraepidermal sensory nerve fibers induced by RTX on nociceptive Schwann cells led us to examine the ultrastructure of these cells. Transmission electron microscopy revealed swollen axons with amorphous or condensed axoplasm, containing granular debris and accumulation of mitochondria, consistent with RTX acting on these unmyelinated axons. However, nociceptive Schwann cells also displayed defects to varying degrees. Some displayed a complete absence of axons with dense, granulated, shrunk, elongated nuclei and absence of plasma membrane ensheathment structures. Others contained swollen axons, displayed shrunk nuclei with axons distally, and only partly ensheathed by nociceptive Schwann cell plasma membrane (Fig. 7).

## 5. Discussion

This study defines nociceptive Schwann cells as critical for maintenance of epidermal nociceptive nerves. Mice deficient of nociceptive Schwann cells display a retraction of epidermal nerve fibers and development of mechanical, cold, and heat hyperalgesia. Conversely, compromising nociceptive nerves such that they retract from epidermis causes a retraction also of the epidermal nociceptive Schwann cell processes. These findings indicate a mutual dependence of the epidermal nerve and the Schwann cell processes covering and abutting these nerves and identifies nociceptive Schwann cells to be critical for epidermal nerve innervation. Thus, the demise of these Schwann cells without directly affecting the nerve can by itself lead to small fiber neuropathy and neuropathic-like pain.

These results might be considered counterintuitive in relation to our previous study where we find nociceptive Schwann cells to contribute to nociceptive mechanical sensation.^1^ If nociceptive Schwann cells contribute to detection of painful mechanical stimuli, the loss of these cells would be expected to result in analgesia instead of hyperalgesia. We believe that this study illustrates the functional importance of the close structural and functional association between nociceptive Schwann cells and nociceptive nerves. The nociceptive glioneural complex involves a mesh-like structure of Schwann cells and nerves, and this structure is encapsulated by cartilaginous fibrillary collagen.^1^ Because the nociceptive Schwann cells represent the end-organ cells, deficits as a result of their demise not only result in epidermal nerve retraction, but also a complete loss of the organ structure. Thus, we believe that the sensitization is a consequence of the loss of the nociceptive glioneural complex, which could participate in development of hyperalgesia by sensitization of spared nerves, similar to that observed in mouse models of peripheral neuropathy, such as the spared nerve injury and sciatic nerve chronic constriction injury models. In the genetic ablation strategy, nociceptive Schwann cell numbers were reduced to close to half that of control mice 7 days after ablation. The normalization of nociceptive Schwann cell numbers and reversal of the pain phenotypes at day 35 after ablation indicates a repopulation/specialization of this cell type either from the remaining spared nociceptive Schwann cells or from Remak Schwann cells.

Out of all intraepidermal nerve fibers of the mouse glabrous and hairy skin, 40% are peptidergic and express CGRP and TRPV1, while the remaining 60% are nonpeptidergic and express MRGPRD but not TRPV1.^46^ Because RTX is a TRPV1 agonist, it stimulates and then inactivates TRPV1-containing nerves that progress to degeneration of the peripheral endings resulting in heat analgesia, as has previously been shown.^15,28^ The RTX model also leads to a paradoxical mechanical allodynia, which could either be explained by sensitization of spared nerve fibers^14,30^ or by central sensitization as a consequence of ligation of TRPV1 in peptidergic fibers by RTX. Our EM results suggest that RTX-induced nerve damage leads to secondary deficits of the nociceptive Schwann cells. This might place sensitization peripherally because nociceptive Schwann cells are not specialized to any particular sensory axon type, but each nociceptive Schwann cell can abut both peptidergic and nonpeptidergic axons.^1^Our results show that the nociceptive Schwann cells are critical for maintaining skin innervation, suggesting that the end-organ Schwann cells signal to the nerve and possibly also provide structural support. Such mechanisms have previously been proposed for the Pacinian and Meissner LTMR end-organs. These studies show that LTMR sensory nerves rely on end-organ Schwann cells during a critical window in development, which reflects a temporary neuronal dependence of neurotrophic factors produced in the target tissue of innervation.^26,36^ Thus, by delaying cutaneous nerve innervation through a nerve crush in neonatal animals, only few LTMR corpuscles can be formed de novo and their morphology and reinnervation is impaired.^43^ GNDF family of ligand signaling through interaction with the RET tyrosine kinase that is expressed by the LTMR neurons innervating Meissner and Pacinian corpuscles confer this effect. In the absence of RET, Pacinian corpuscles do not form and Meissner corpuscles are disorganized morphologically.^4,23^ These sensory organ deficits are not caused by the death of neurons, but likely a result of failed differentiation and target innervation due to lack of the trophic signaling and as a result, the sensory organs do not develop because they lack the nerve, which induces their development. A similar dependence could explain the loss of epidermal innervation in the Schwann cell-ablated mice. Luo et al.^23^ found that epidermal innervation of nociceptors is almost completely lost after deletion of RET in sensory neurons of young adult mice, showing that a continuous RET signaling is required for maintenance of intraepidermal nerves. The importance of terminal Schwann cells for nerve endings has been studied in greater detail in the neuromuscular junction where the end-organ Schwann cell processes can extend in the absence of axons and determine axon morphology and reinnervation.^33^ Furthermore, nerve retraction and functional deficits are observed after perturbing specifically the perisynaptic Schwann cells in the adult.^32^ Thus, the neuromuscular junction, similar to nociceptors, relies on terminal Schwann cells for axon terminal maintenance in the adult, and in their absence, axons retract.

Axons also depend on a metabolic support from Schwann cells.^13,27^ This metabolic support maintains axonal integrity and consequently, mitochondrial dysfunction in Schwann cells has been proposed to contribute to peripheral neuropathies.^9,27,29^ Mice with an impaired mitochondrial function by deletion of mitochondrial transcription factor A (Tfam) in embryonic Schwann cells causes degeneration of preferentially unmyelinated axons.^37^ Similar results have been reported in mice with embryonic ablation of the serine/threonine kinase LKB1, also involved in mitochondrial metabolism.^2,31^ Schwann cell metabolism is continuously critical for maintenance of unmyelinated nerves because deleting Lkb1 in adult Schwann cells also result in axon degeneration and peripheral neuropathy.^2^ Thus, compromising metabolism in Schwann cells leads to degeneration of unmyelinated axons and behavioral symptoms of painful neuropathy. However, at the time of these studies, the association in the skin of intraepidermal nerves with nociceptive Schwann cells was not known and hence, the genes were deleted in all Schwann cells. Considering that we ablate the final tip Schwann cell (ie, the nociceptive Schwann cell), leaving most other Remak Schwann cells intact, it seems unlikely that the nerve deficits observed are caused solely by a failure of metabolic support.

We also find that the nociceptive Schwann cells critically depend on the nerve, similar to earlier studies on some LTMR end-organs. Organogenesis during development of Meissner corpuscles, Pacinian corpuscles, Golgi tendon organs, and muscle spindles critically depends on the nerve.^8,42^ In the Schwann cell sensory organs, the nerve supports Schwann cell survival and differentiation by providing the growth factor Neuregulin 1 type III, which interacts with the Neuregulin receptors ErbB2 and ErbB3 expressed by the Schwann cells. Consistently, exogenous delivery of Neuregulin 1 prevents end-organ Schwann cell apoptosis of Pacinian corpuscles during development.^20^ Remak Schwann cells also rely on Neuregulin 1; consequently, nociceptive nerves lacking Neuregulin 1 have defective Remak Schwann cell ensheathment,^12^ and this deficit is not compensated by any other mechanism.^11^ Thus, if nociceptive Schwann cells share mechanisms with Pacinian sensory end-organ Schwann cells and Remak Schwann cells, the severe consequences observed in this study on nociceptive Schwann cells by chemical ablation of nerves could indicate a continuous dependence of nociceptive Schwann cells on Neuregulin 1 supplied by nociceptive nerves. In this case, such dependence would differ from adult Pacinian corpuscles, which survive denervation without effects on corpuscle numbers or morphology.^10,44^

The interdependence of the nerve and the nociceptive Schwann cell precludes any conclusion on the etiology in patients with peripheral neuropathy. However, we conclude that in mice, demise of nociceptive Schwann cells is sufficient by itself for the manifestation of small fiber neuropathy and neuropathic pain, which warrants for further studies to explore if known small fiber neuropathy, such as those caused by chemotherapy or by treatments leading to diabetic neuropathy, could also be caused by a damage to nociceptive Schwann cells.

## Conflict of interest statement

The authors have no conflicts of interest to declare.
